# Precipitation Behavior and Properties Evolution of Cu-1.16Ni-0.36Cr Alloy During Heat Treatment

**DOI:** 10.3390/ma18163885

**Published:** 2025-08-19

**Authors:** Shaolin Li, Shuaibin Li, Wenming Sun, Qiangsong Wang, Kexing Song

**Affiliations:** 1 Henan Key Laboratory of Nonferrous Materials Science and Processing Technology, Henan University of Science and Technology, Luoyang 471023, China; 240320020214@stu.haust.edu.cn (S.L.); 18848967913@163.com (W.S.); 2 Institute of Materials, Henan Academy of Sciences, Zhengzhou 450046, China; wangqiangsongbj@163.com; 3 State Key Laboratory of Nonferrous Metals and Processes, GRINM Group Co., Ltd., Beijing 100088, China; 4 GRIMAT Engineering Institute Co., Ltd., Beijing 101407, China

**Keywords:** Cu-1.16Ni-0.36Cr alloy, aging treatment, microstructure, strengthening mechanism

## Abstract

In this paper, Cu-1.16Ni-0.36Cr alloy was obtained by adding Ni-Cr intermediate alloy, and the effects of aging parameters on its microstructural evolution and mechanical properties were studied. The results show that after secondary aging (solid solution + one time cold rolling at 87.5% + annealing at 300 °C for 2 h + secondary aging at 450 °C for 2 h), dispersed BCC structure Cr precipitates are obtained in the alloy, which shows good comprehensive properties (strength of 512.0 MPa, elongation of 17.2%, and electrical conductivity of 45.5%). The change in aging parameters significantly affects the existing form of Cr precipitates. When the aging temperature increases from 400 °C to 450 °C, the precipitated phase begins to have a stable boundary, which shows that the precipitated phase with BCC structure Cr precipitates (~10 nm in range). When the aging temperature further increases to 500 °C, the size of Cr precipitated phase begins to grow, from 5.0 nm to 16.7 nm. The strengthening mechanism of the alloy with different aging time at 450 °C is calculated, and the relationship among aging parameters, microstructure characteristics, strengthening mechanism and mechanical properties is established. It is concluded that precipitation strengthening and dislocation strengthening are the main strengthening mechanisms of the alloy.

## 1. Introduction

After aging treatment, the Cr element in the Cu-Ni-Cr alloy will precipitate in the form of precipitated phase, strengthening the alloy through second-phase strengthening. Meanwhile, there is a phenomenon of modulated decomposition, which reduces the existence of grain boundaries. This alloy is extensively employed in pipeline valves, marine condensation pipes, etc. [1,2,3]. When the content of Cr element is relatively high, there will be enrichment of Cr element at the grain boundaries, resulting in a decrease in the mechanical properties of the alloy [4]. This alloy system has both good mechanical properties and high-temperature properties, and also has good application prospects in the field of electronic materials. The composition design and application scenarios of the alloy have been the focus of research on this alloy.

Since the introduction of Cu-Ni-Cr alloys, research has primarily focused on alloy composition design, phase diagram calculations, and spinodal decomposition [5,6,7]. In recent years, low-solubility Cu-Ni-Cr alloys have garnered increasing attention for electronic applications owing to their excellent thermal and corrosion resistance, non-magnetic nature, and mechanical strength. Wang et al. [8] discovered that a Cu-0.27Cr-0.19Ni alloy exhibits amplitude modulation decomposition after aging treatment, with favorable high-temperature stress relaxation properties. Cheng et al. found that Cu-Ni-Cr alloys maintain good mechanical properties when possessing favorable thermoelectric characteristics. Trivedi et al. [9] found that Cu-Cr-Ni alloys used as electrical contact electrode materials exhibit no significant property degradation after exposure to 723 K for 15 days.

In recent years, Cu-Ni-Cr alloys have been extensively studied for their applications in electronic materials. They exhibit excellent mechanical and electrical properties, as well as superior high-temperature service performance, machinability, and weldability, showing great prospects in the field of lead frame materials. Research shows that Cr has low solubility in copper alloys. Aging treatment can strengthen the alloy through precipitation, but Cr precipitates tend to coarsen and aggregate at high temperatures, limiting the alloy’s high-temperature applications. Hindering the growth of Cr precipitates is a key way to improve this alloy system [10,11,12]. Studies have found that large cold deformation and aging treatment can promote the dispersion and precipitation of precipitates. Nano-sized precipitates can significantly enhance the mechanical properties of the alloy and prevent coarsening at high temperatures, thus strengthening the alloy’s performance. Adding intermediate alloys can help Cr fully integrate into the copper matrix, enabling its dispersed precipitation. Research indicates that Ni and Cr can form Ni2Cr ordered phases [13,14,15] and NiCr metastable phases [16]. A Ni/Cr ratio between 2.5 and 3.5 is conducive to the formation of intermediate phases. Currently, there is limited research on deformation heat treatment strengthening of Cu-Ni-Cr alloys and on adjusting their properties via aging parameters. Studying the microstructural evolution during aging is significant for the development and application of these alloys.

This paper takes Cu-Ni-Cr alloy as the research object, introducing alloying elements by adding Ni/Cr master alloys while controlling the Ni/Cr ratio at 3. The authors adjust the alloy’s properties by optimizing the aging treatment parameters during mechanical thermal treatment, investigating the effects of aging process parameters on the alloy’s microstructure and properties, which is of great significance for the development and application of new alloys.

## 2. Materials and Methods

The initial billet is obtained by melting the raw materials using a medium-frequency induction furnace. The raw materials consist of high-purity copper (99.99%) and Ni-Cr intermetallic alloy. During melting, the crucible is preheated on a resistance wire, followed by the addition of high-purity copper. Once the copper is completely melted, the Ni-Cr intermetallic alloy is added. The mixture is stirred using a high-purity graphite rod, with charcoal used as a cover agent. After thorough stirring and heat preservation, the alloy solution is poured into a high-purity graphite crucible (Φ80 mm × 200 mm) to complete the casting process. The melting temperature is maintained at 1200–1300 °C, while the pouring temperature is controlled at 1100–1150 °C. To eliminate segregation of alloying elements, the as-cast alloy is subjected to a homogenization annealing process at 950 °C for 12 h. The nominal composition of the alloy obtained from melting is determined to be Cu-1.16Ni-0.36Cr.

**Figure 1:**
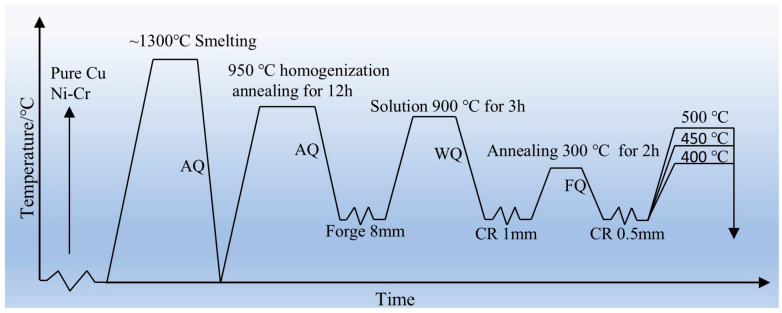
Deformation and heat treatment process. AQ (air quenching), WQ (water quenching), CR (cold rolling), FQ (furnace quenching). Adapted from Ref. [17].

Figure 1 [17] illustrates the processing route of the alloy. Through previous work, the optimal hot-working parameters for this alloy were determined to be 950 °C/1s^−1^ and the alloy ingot was forged into plates with dimensions of 200 mm × 160 mm × 8 mm. The as-forged alloy underwent solution treatment at 900 °C for 3 h, and the cooling mode was water cooling. Subsequently, the alloy plates were cold-rolled with a deformation of 87.5%, reducing the thickness to 1 mm. Annealing treatment was then performed to eliminate dislocations accumulated in the alloy due to heavy cold deformation; the annealing temperature was 300 °C, the time was 2 h, and the cooling mode was air cooling. After annealing, the alloy underwent secondary cold rolling. Previous research indicated that a 50% deformation during secondary cold rolling yielded the optimal comprehensive properties, resulting in an alloy thickness of 0.5 mm. The cold-rolled thin plates were then subjected to aging treatment to investigate the effects of aging process parameters on alloy properties. The aging temperatures were 400 °C, 450 °C, and 500 °C, with aging times of 0.5 h, 1 h, 2 h, 3 h, and 4 h.

Tensile tests were conducted at room temperature using a WDW-100D (Jinan Kairui Testing Machine Manufacturing Co., Ltd., Jinan, Shandong, China) precision universal testing machine at a strain rate of 1.0 × 10^−3^ s^−1^, and the tests were repeated three times. The tensile specimens were machined into dog-bone shapes (width of 10 mm and length of 30 mm) in accordance with the national standard GB/T 228.1-2010. The electrical conductivity was measured at room temperature using a Sigma 2008 B1 (Sigma Aldrich, St. Louis, MO, USA) digital eddy-current metal conductivity meter (Φ16 mm). Five measurements were taken at different positions and averaged. The microhardness of the alloy at room temperature was measured using an HVS-1000A digital hardness tester with a load of 100 g applied for 15 s. The XRD patterns of the alloy were obtained using a Bruker D8 X-ray diffractometer (Biflerica, MA, USA) with a scanning range of 40° to 100°, a step size of approximately 0.02°, and a scanning speed of approximately 4°/min. The microstructure of the alloy was analyzed using an in-verted metallurgical microscope (DMi8C, Leica Microsystems Inc., Wetzlar, Germany) and a scanning electron microscope (SEM, JSM-5610LV. JEOL, Tokyo, Japan)

Using a JEOL JSM-7800F field-emission scanning electron microscope (JEOL Ltd., Tokyo, Japan) to acquire sample information, the specimen was tilted to 70° and the accelerating voltage was set to 20 kV to maximize the hit rate. Using a JSM-2100 transmission electron microscope (JEOL Ltd., Tokyo, Japan) to observe the alloy, the sample was mechanically ground and polished to a thickness of 50–70 μm, punched into 3 mm disks, and then thinned to electron transparency using a Gatan 659 precision ion-polishing system (Gatan, Pleasanton, CA, USA).

## 3. Results

### 3.1. Subsection Evolution of Electrical Conductivity and Mechanical Properties of Cu-1.16Ni-0.36Cr Alloy During Aging Treatment

**Figure 2:**
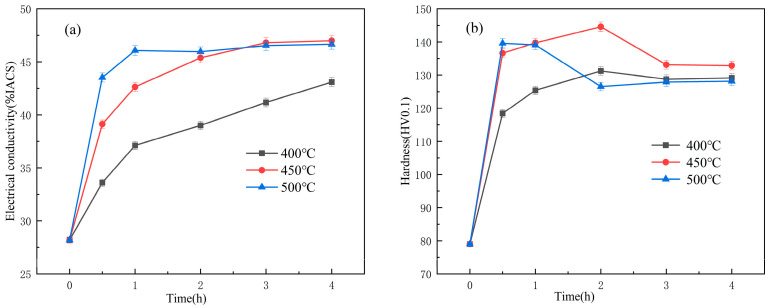
Variation curves of electrical conductivity and hardness of Cu-1.16Ni-0.36Cr alloy under different aging parameters: (**a**) the relationship between electrical conductivity and time; (**b**) the relationship between hardness and time.

Figure 2 presents the variation curves of hardness and electrical conductivity of the Cu-1.16Ni-0.36Cr alloy under different aging parameters after solution treatment and secondary cold rolling. Figure 2a shows the variation curves of electrical conductivity of the alloy aged at 400 °C, 450 °C, and 500 °C as the aging time increases, indicating that the aging process significantly enhances the electrical conductivity of the alloy. As the aging time increases, the electrical conductivity shows an overall upward trend under all three temperature conditions, with higher aging temperatures leading to faster increases in electrical conductivity. When the aging time reaches 3 h, the electrical conductivity of the alloy tends to stabilize. Figure 2b illustrates the variation trend of alloy hardness with aging time under different aging temperatures. It can be observed that the aging process has a significant hardening effect on Cu-1.16Ni-0.36Cr alloy, with substantial strength improvement in the early aging stage. Specifically, at 400 °C aging temperature, the alloy reaches a peak hardness of 131.1 HV0.1 after 2 h of aging; at 450 °C, it reaches 144.4 HV0.1 after 2 h; and at 500 °C, it reaches 139.8 HV0.1 after 0.5 h. With further increase in aging time, the hardness of the alloy decreases. As the aging temperature increases, the peak hardness of the alloy first increases and then decreases, reaching the maximum value of 144.4 HV0.1 at 450 °C aging temperature for 2 h.

**Figure 3:**
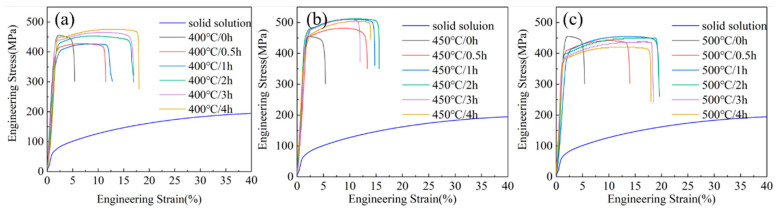
Stress–strain curves of Cu-1.16Ni-0.36Cr alloy with different aging parameters. (0 h indicates annealing and cold working): (**a**) 400 °C with different aging times; (**b**) 450 °C with different aging times; (**c**) 500 °C with different aging times.

Figure 3 presents the typical engineering stress–strain curves of Cu-1.16Ni-0.36Cr alloy under different aging parameters after solution treatment and secondary cold rolling, and Table 1 presents the tensile strength and elongation values of Cu-1.16Ni-0.36Cr alloy under different aging parameters. After solution treatment, the alloy strength is 196.7 MPa, and the elongation is 49.7%. After cold rolling–annealing–secondary cold rolling, the alloy strength increases significantly, while the elongation decreases sharply. At this time, the strength is 454.7 MPa without aging treatment, and the elongation is only 5.4%. After aging treatment, the strength increases, and the elongation improves significantly. As shown in Figure 3a, after aging treatment at 400 °C, the tensile strength decreases slightly after short-time aging, and, with the increase in aging time, the strength increases significantly, and the elongation remains stable at around 17.5%. As shown in Figure 3b, after aging treatment at 450 °C, the strength increases more significantly, and, with the aging time increasing to 2 h, the strength reaches 512 MPa, with the elongation at 17.2%. As the aging time further increases, the strength remains stable, while the elongation decreases rapidly. When the alloy is aged at 500 °C, the yield strength decreases, the tensile strength is similar to that of the unaged state, and the elongation increases significantly. It can be seen that for Cu-1.16Ni-0.36Cr alloy, after aging treatment, the elongation increases significantly, while the strength does not improve obviously. It can also be found that the aging temperature has a greater impact on the properties of the alloy. At 400 °C, the aging is insufficient, and at 500 °C, the alloy undergoes softening, which is a typical characteristic of over-aging.

### 3.2. Microstructure of Solid Solution Cu-1.16Ni-0.36Cr Alloy

**Figure 4:**
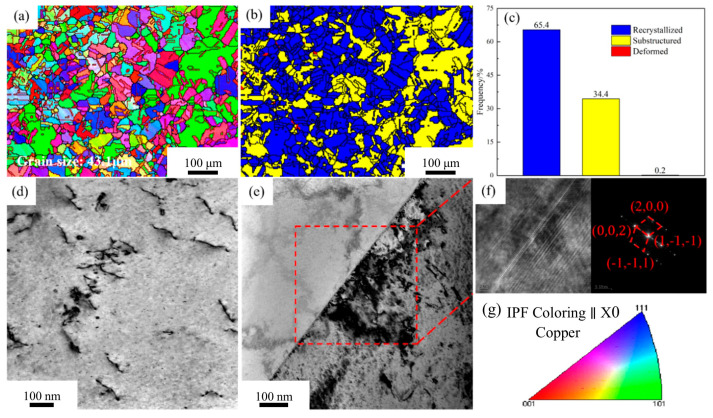
EBSD images and TEM images of Cu1.16Ni-0.36Cr alloy in the solution-treated state: (**a**) OIM chart; (**b**) recrystallization degree chart; (**c**) recrystallization statistics; (**d**,**e**) TEM images; (**f**) high-resolution image of the region in figure; (**g**) with the corresponding FFT image.

Figure 4 presents the microstructure of the Cu-1.16Ni-0.36Cr alloy in the solution-treated state. Figure 4a shows the orientation imaging map (OIM) of the solution-treated alloy. High-angle grain boundaries (HAGBs) with misorientation angles over 15° are marked with thick solid lines. Low-angle grain boundaries (LAGBs) with angles between 2° and 15° are indicated with thin solid lines. The inverse pole figure (IPF) color scheme is presented in Figure 4g. The image reveals that after solution treatment, the alloy consists of equiaxed grains with uniform size and orientation. Additionally, numerous annealing twins generated by rapid cooling are present. Twin boundaries are highlighted with red solid lines in the figure. The average grain size within the alloy at this point is 43.1 μm. Figure 4b statistically analyzes the recrystallization fraction of the alloy. It is found that the alloy has nearly fully recrystallized. The recrystallization fraction reaches 65.4%. Meanwhile, a small amount of substructure exists. This indicates that the solution treatment parameters are appropriate. Figure 4d is the transmission electron microscopy (TEM) image of the solution-treated alloy. It is observed that after solution treatment, only a few linear defects are uniformly distributed in the alloy matrix. There are almost no other defects. Annealing twins within the alloy are detected via TEM. Figure 4e presents the TEM image of the annealing twin structure. This is consistent with the observations in Figure 4a. Figure 4f display the high-resolution images of twin boundaries and the corresponding fast Fourier transform (FFT) patterns. They are indexed as the Cu phase.

### 3.3. Microstructure Evolution of Cu-1.16Ni-0.36Cr Alloy During Aging

#### 3.3.1. EBSD Analysis of Microstructure Evolution of Alloy During Aging

**Figure 5:**
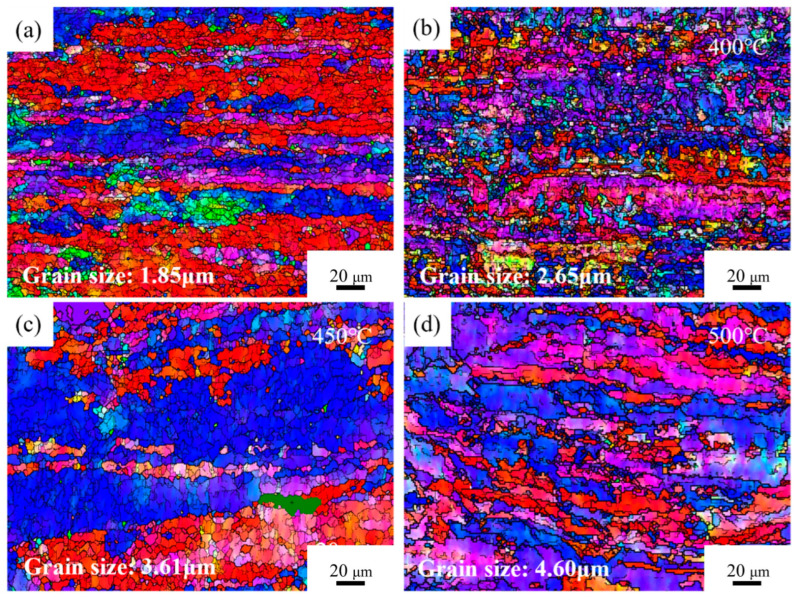
OIM of Cu1.16Ni-0.36Cr alloy in different states: (**a**) cold-rolling state; (**b**–**d**) aging state.

Figure 5 presents the OIM maps of Cu-1.16Ni-0.36Cr alloy under different conditions. Figure 5a shows the OIM map of the cold-rolled alloy without aging treatment after secondary cold rolling. The results indicate that after double-step cold rolling, the grains in the alloy were significantly refined, exhibited distinct orientation, and were distributed in an elongated shape, with an average grain size of 1.85 μm. The staining results show that a large number of grains parallel to the <001> orientation were generated at this time. Figure 5b presents the OIM map of the aged alloy after 2 h at 400 °C. The results show that a large number of fine equiaxed grains were generated inside the alloy, and the deformed grains parallel to the <001> direction were significantly reduced and replaced by grains parallel to the <111> direction. There were still a large number of small-angle grain boundaries and large deformed grains inside the alloy, and the average grain size inside the alloy was 2.65 μm at this time. Figure 5c is the OIM map of the aged alloy after 2 h at 450 °C, and the image shows that the directionality of the grains inside the grains was significantly eliminated, a large number of clustered equiaxed grains began to appear, and the large deformed grains were gradually replaced by equiaxed grains with uniform size. Figure 5d shows the OIM map of the alloy aged for 2 h at 500 °C. At this time, the alloy’s grain boundaries were distinct and the grains had grown significantly. The average grain size within the alloy was 4.60 μm. The crystal interior overall showed uniform elongated grains, with almost no large deformed grains visible.

**Figure 6:**
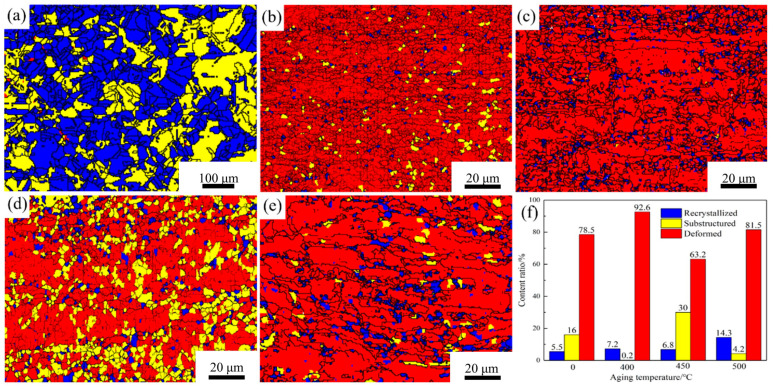
Recrystallization diagrams and recrystallization statistics of Cu-1.16Ni-0.36Cr alloy in different states: (**a**) recrystallization diagram of an alloy in solid solution; (**b**) cold-rolling state; (**c**–**e**) aging state; (**f**) recrystallized grain statistics.

Figure 6 presents the recrystallization images and grain statistics of the Cu-1.16Ni-0.36Cr alloy under different heat treatment parameters. Figure 6a shows the recrystallization diagram of the solution-treated alloy. Figure 6b indicates that after secondary cold rolling, the alloy interior is almost entirely composed of deformed grains, with only a few subgrains present. After 400 °C aging treatment, a small number of recrystallized grains appear, with the grain transformation primarily manifested as the conversion of subgrains to recrystallized grains, as shown in Figure 6c. At 450 °C, the recrystallized grains in the alloy interior remain essentially stable, while subgrains significantly increase. As the temperature rises further, the number of recrystallized grains markedly increases from 6.8% to 14.3%, with a concurrent significant reduction in subgrains. It can be concluded that after secondary cold rolling, the alloy interior is almost entirely occupied by deformed grains, with only a few dynamic recrystallized grains and subgrains present. In the initial stage of recrystallization, the subgrains generated during cold rolling rapidly transform into recrystallized grains. However, due to the low temperature, the transformation efficiency of deformed grains to substructures is relatively low. When the temperature reaches 450 °C, both the rate of subgrain conversion to recrystallized grains and the rate of subgrain generation rapidly increase, with the rates being comparable. As the temperature rises further, the recrystallization rate rapidly increases, leading to a significant rise in recrystallized grain quantity and a swift decrease in subgrains. At this point, the primary transformation mode of alloy recrystallization is also the conversion of subgrain grains to recrystallized grains. The maximum recrystallization extent achieved at this point is 14.3%. It is evident that this alloy has a strong ability to resist the transformation of deformed grains into recrystallized grains at high temperatures.

**Figure 7:**
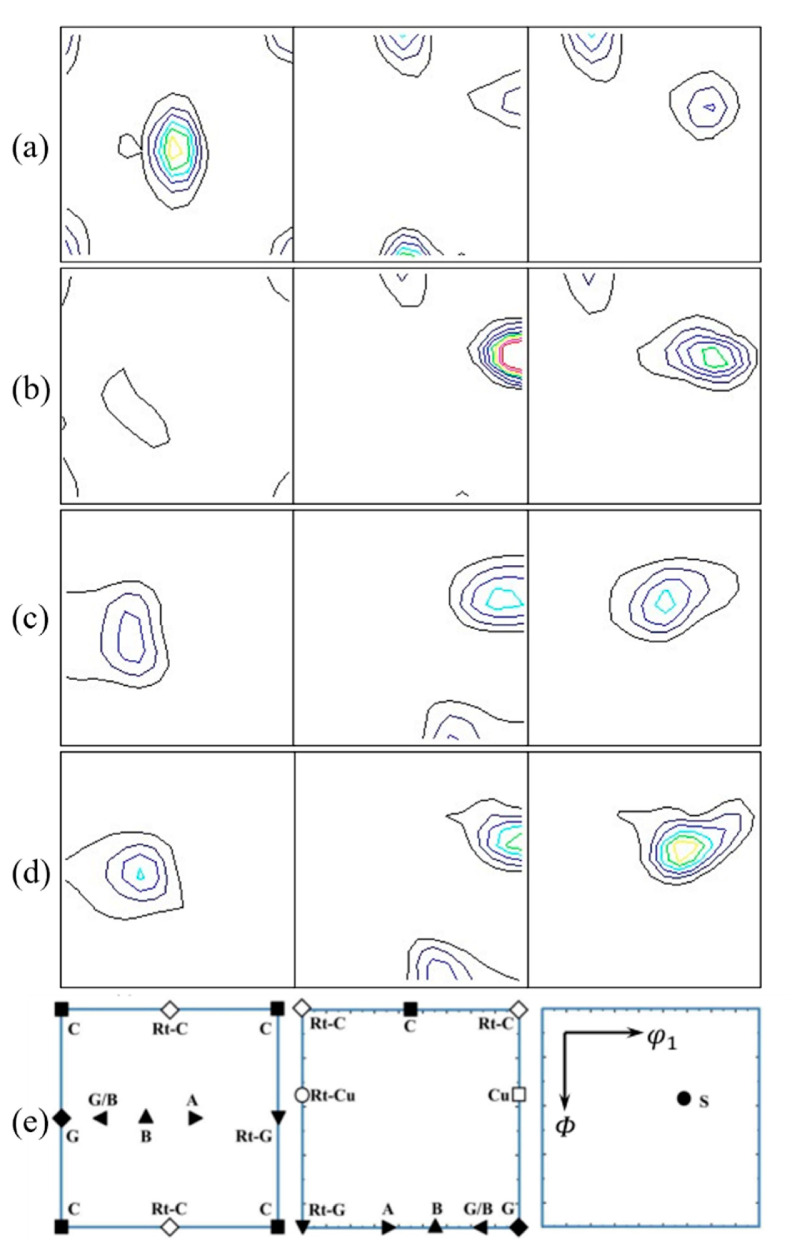
ODF diagram of Cu-1.16Ni-0.36Cr alloy at 0°, 45°, 65° in different states and typical preferred orientation of FCC structure: (**a**) cold-rolling state; (**b**–**d**) aging time for 400 °C, 450 °C, 500 °C aging 2 h for aging state alloy; (**e**) Orientation diagram.

Figure 7 presents the ODF maps of the Cu-1.16Ni-0.36Cr alloy in the cold-rolled state and after different aging temperatures, for sections at 0°, 45°, and 65°. The typical preferred orientations of face-centered cubic metals after rolling are shown in Figure 7e. The texture statistics within the alloy are presented in Table 2. From Figure 7 and Table 2, it can be concluded that after cold rolling, a high texture intensity is generated, with the cold-rolled texture mainly consisting of S, Copper, and Brass textures. After aging treatment, the types of textures in the alloy remain largely unchanged, but the intensity of the textures varies, with significant changes in the S and Copper textures. As the aging temperature increases, the S texture decreases significantly, while the intensity of the Copper texture increases slightly. The orientation of the S texture is <123> parallel to the plate surface. During cold rolling, its intensity rises rapidly, making it the dominant texture in the rolling process. During aging, deformed grains undergo partial recrystallization, reducing the intensity of the S texture. When the temperature rises to 500 °C, the intensity of the S texture drops sharply, indicating a significant reduction in the number of elongated grains along the deformation direction within the alloy. As the aging temperature increases, the intensity of the Copper texture shows an overall upward trend, corresponding to the promotion of the increase in <111>-oriented grains during aging, as shown in Figure 3. The Brass texture decreases slightly after aging treatment, reflecting the disappearance of slip deformation within the alloy during aging, with the most significant change occurring at 450 °C.

#### 3.3.2. TEM Analysis of Alloy During Aging

**Figure 8:**
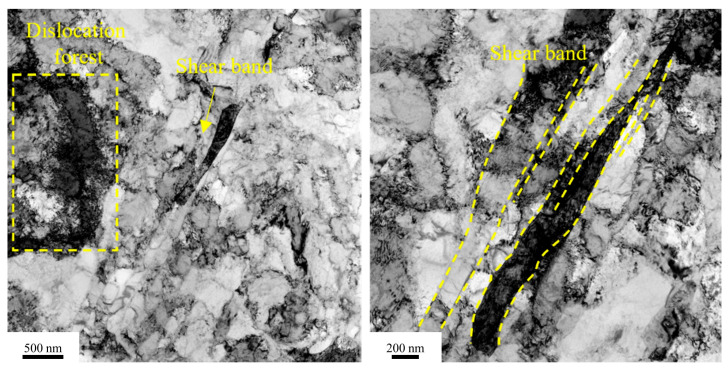
TEM image of cold-rolled alloy.

Figure 8 shows the transmission electron microscopy (TEM) image of the cold-rolled Cu1.16Ni-0.36Cr alloy. After secondary cold rolling, the grains were crushed, and the grain size was significantly reduced. The cold-rolling process generated a large number of dislocations within the alloy, which accumulated and began to tangle. There were numerous nanoscale lamellar grains, forming a deformation band. The slip band direction was along the grain elongation direction, with a width of 100–200 nm. No precipitates were observed in the gaps between the slip bands.

**Figure 9:**
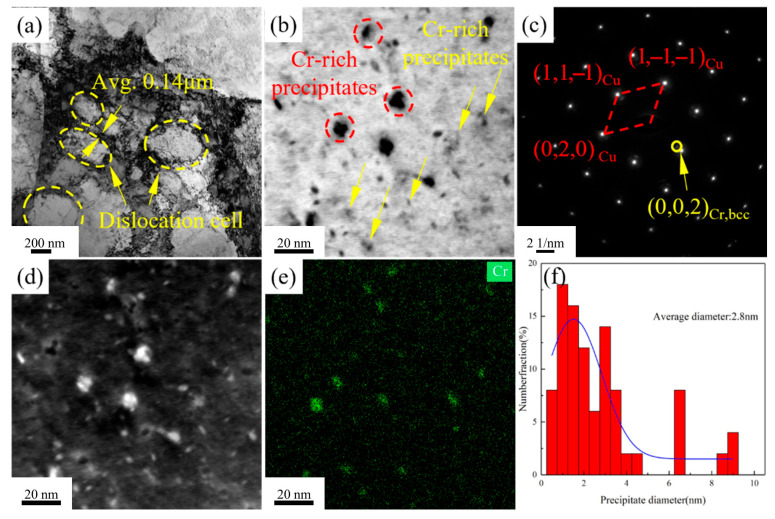
TEM image of Cu-1.16Ni-0.36Cr alloy aged at 400 °C for 2 h: (**a**) substructure diagram of dislocation cell; (**b**) distribution of Cr-enriched precipitation phases; (**c**) electron diffraction pattern for this selection; (**d**,**e**) HAADF-TEM scanning image; (**f**) dimensional statistics of precipitated phases in alloys.

Figure 9 presents the TEM images of Cu-1.16Ni-0.36Cr alloy after aging at 400 °C for 2 h. Figure 9a shows that, after the process of secondary cold rolling combined with aging treatment, dislocations in the Cu-1.16Ni-0.36Cr alloy begin to aggregate and entangle, forming a large number of dislocation cell substructures. The average size of equiaxed dislocation cells is 0.35 μm, with dislocation cell wall thicknesses of 0.14 μm. Figure 9b indicates that numerous nanoscale precipitates form in the alloy after aging treatment. Two main types of precipitates exist in the alloy matrix: granular precipitates and pod-shaped precipitates. Figure 9d,e show HAADF-TEM scans of the alloy, revealing that both types of precipitates are located in Cr-enriched regions, confirming them as two distinct Cr-based precipitate phases. Figure 9c presents selected-area electron diffraction patterns from this region, showing diffraction spots from the BCC-structured copper matrix and adjacent diffraction spots from the BCC-structured Cr phase. Statistical analysis of precipitate sizes reveals an average diameter of 2.8 nm for precipitates dispersed in the matrix at this stage, with Figure 9f presenting the size distribution statistics of precipitates within the alloy.

**Figure 10:**
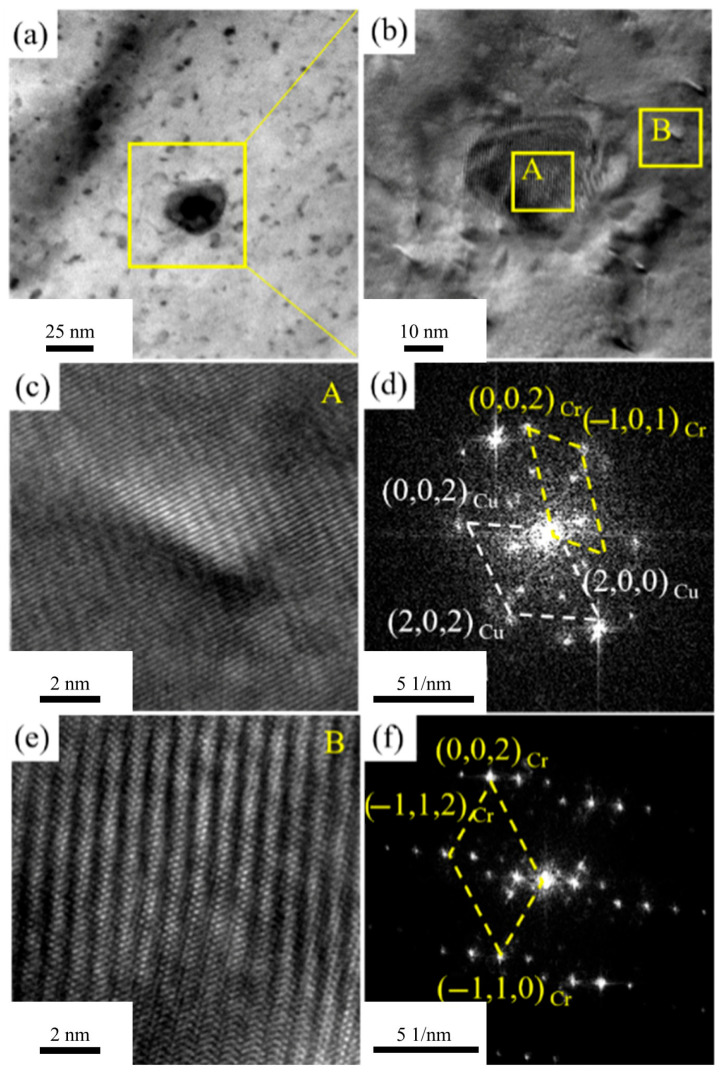
TEM images of the second phase in Cu-1.16Ni-0.36Cr alloy aged at 400 °C for 2 h: (**a**) bright-field image of the precipitated phase inside the alloy; (**b**) HRTEM of the precipitated phase in Figure 10a; (**c**) HRTEM of region A in Figure (**b**); (**d**) electron diffraction pattern for region A; (**e**) HRTEM of region B in Figure 10b; (**f**) electron diffraction pattern for region B.

To further investigate the structure of Cr-rich precipitates formed during aging of the Cu1.16Ni-0.36Cr alloy, Figure 10 presents TEM and high-resolution images of the alloy aged at 400 °C for 2 h. Figure 10a shows numerous precipitates within the alloy. HRTEM images of the precipitate-aggregated area in Figure 10a are shown in Figure 10b, revealing two types of precipitates: larger ones of around 10 nm with a regular shape and distinct boundaries, and smaller ones of around 1 nm with indistinct boundaries. HRTEM observations of the two precipitates are shown in Figure 10c,d, with FTT processing in Figure 10d,f. Small precipitates are irregularly shaped and fully coherent with the Cu matrix. Two sets of diffraction spots are observed: FCC Cu matrix and BCC Cr precipitates. The orientation relationship is (11-1)Cu ∥ (002)Cr and [110]Cu ∥ [010]Cr. Figure 10f shows large precipitates have BCC Cr diffraction spots. Secondary diffraction spots around Cr phases match the BCC structure and correspond to moiré fringes in HR images. Cr phases are semi-coherent with the Cu matrix. Precipitate formation reduces solute atom electron scattering, significantly increasing electrical conductivity after aging. The dispersed precipitates in the matrix also significantly enhance alloy strength. Cr precipitates do not transform from FCC to BCC, likely due to cold deformation processing. Significant cold deformation promotes nucleation and growth of precipitates.

**Figure 11:**
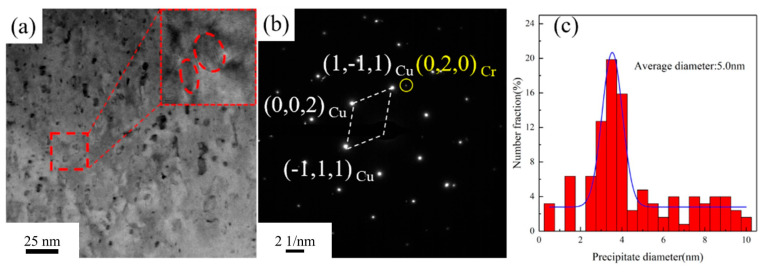
TEM image of Cu-1.16Ni-0.36Cr alloy after aging at 450 °C for 4 h: (**a**) HRTEM of alloys (the upper right corner is a partial enlargement of the red frame); (**b**) diffraction pattern of the red square in Figure 11a; (**c**) average size statistics of precipitated phases.

Figure 11 presents the TEM images of the Cu-1.16Ni-0.36Cr alloy after aging at 450 °C for 2 h. After aging at 450 °C for 2 h, the number of precipitates within the alloy significantly increases. Further analysis of the small-sized precipitates using HRTEM reveals that, compared with those in Figure 10, the small-sized precipitates lose their moiré pattern structure, transforming into granular shapes with a more regular structure. Simultaneously, they gradually lose coherency with the matrix. Calculations show that the lattice parameter of the precipitates is 0.236 nm, while that of the matrix is 0.223 nm, resulting in a lattice mismatch of 7.9% between the precipitates and the matrix. Selected-area electron diffraction (SAED) reveals the BCC Cr precipitates with a clear structure, as shown in Figure 11b. The orientation relationship between the Cr precipitates and the matrix is (002)Cu ∥ (020)Cr and [110]Cu ∥ [100]Cr. As the aging time increases, the size of the precipitates shows significant growth. Statistical analysis of the precipitate sizes in Figure 11a indicates an average size of 5.0 nm for the precipitates.

**Figure 12:**
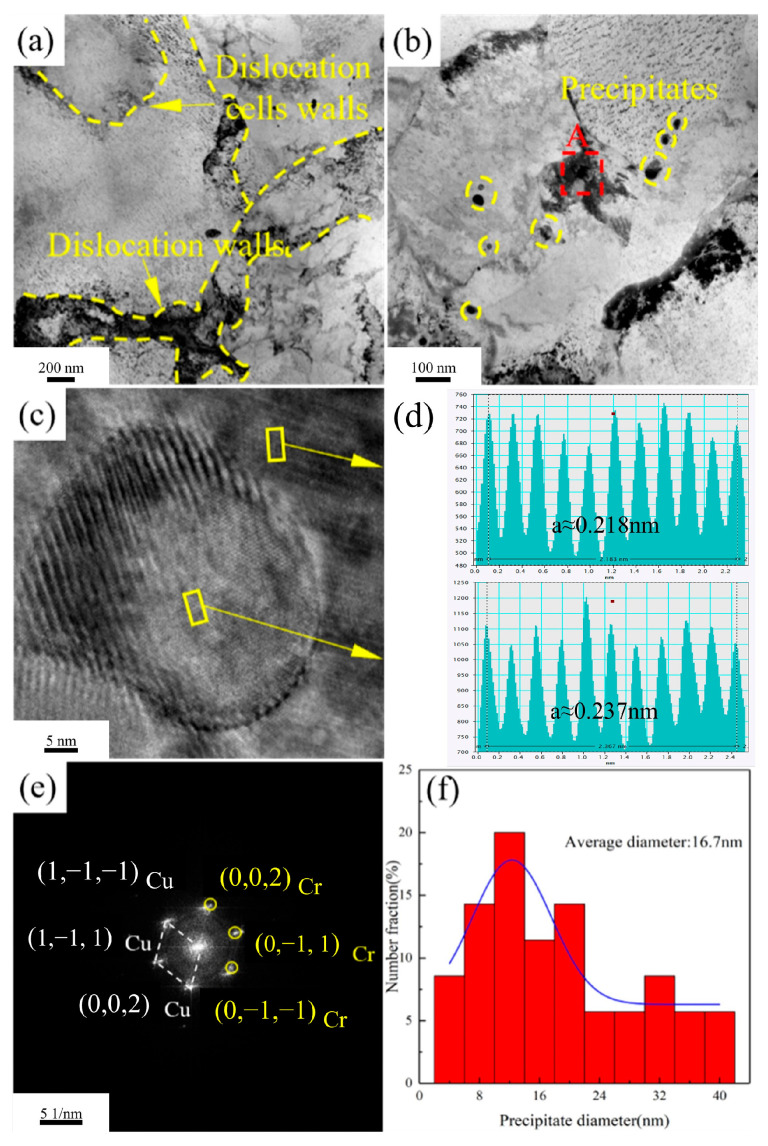
TEM image of Cu-1.16Ni-0.36Cr alloy aged at 500 °C for 2 h: (**a**) distribution characteristics of bright-field images of dislocation cells and dislocation walls in alloys (the red area here is the main area for observation); (**b**) morphology and distribution characteristics of precipitates in alloys; (**c**) HRTEM for precipitated phases (it is the magnification of the red area in Figure 12b); (**d**) intergranular spacing analysis of precipitated phase and matrix; (**e**) electron diffraction pattern of a selection; (**f**) average size statistics of precipitated phases.

Figure 12 shows the TEM images of Cu-1.16Ni-0.36Cr alloy aged at 500 °C for 2 h. From Figure 12a, it can be observed that after aging at 500 °C for 2 h, the dislocations in the alloy have significantly decreased, the size of the formed dislocation cells has increased, and a large number of precipitates have appeared near the dislocation walls, with the precipitate size being larger than that of the matrix. From Figure 12b, it can be seen that the precipitates in the alloy are regularly shaped spherical precipitates, with some clustering at the grain boundaries. HRTEM analysis of the granular precipitates shows that the boundary between the precipitates and the matrix is distinct, and the coherency between the precipitates and the matrix has further disappeared. Measurements show that at this point, the interplanar spacing of the matrix is 0.218 nm, and that of the precipitates is 0.237 nm. Using Formula (1), the misfit of the precipitates is calculated to be 8.35%, and the matrix and precipitates remain in a semi-coherent state. In the selected-area electron diffraction, two complete sets of diffraction spots can be seen, corresponding to the FCC-structured copper matrix and the BCC-structured Cr phase. After aging at 500 °C for 2 h, the size of the precipitates in the alloy further increases. Statistical analysis of the precipitate sizes in the alloy shows that, at this point, the precipitate size has increased to 16.8 nm. As the aging temperature increases, the size of the precipitates in the alloy grows. When the aging temperature increases from 400 °C to 450 °C, the number of precipitates significantly increases, with a marked increase in regularly shaped granular precipitates, and the precipitate size also increases. At an aging temperature of 500 °C, the precipitates in the alloy transform into numerous granular precipitates, with significant coarsening of the precipitate size, which is an important reason for the rapid decrease in alloy strength.

(1) $$\delta =\frac{2\left(\alpha_{\beta }-\alpha_{\alpha }\right)}{\alpha_{\beta }+a_{\alpha }}\times 100\%$$

where $a_{\alpha }$ and $\alpha_{\beta }$ are the lattice constants of the matrix and the precipitated phase with the matrix, respectively.

**Figure 13:**
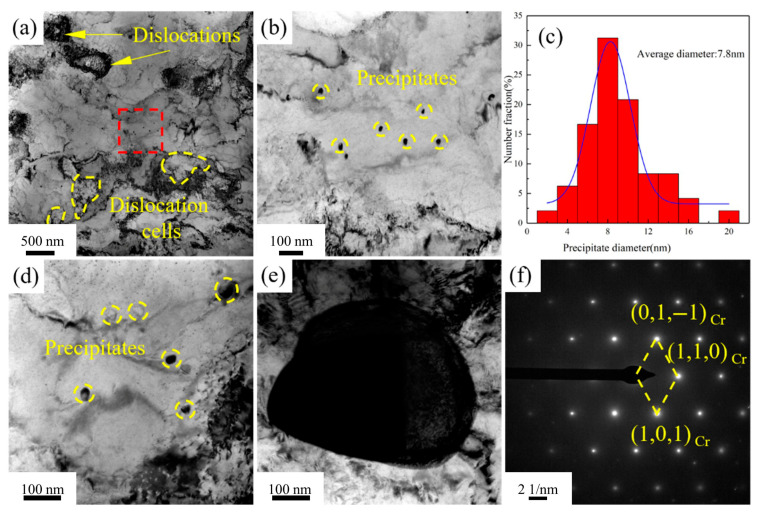
TEM images of Cu-1.16Ni-0.36Cr alloy aged at 450 °C for 4 h: (**a**) distribution of dislocations and dislocation cells (the red area here is the main area for observation); (**b**,**d**) TEM image of the precipitated phase (Figure 13b is the magnification of the red area in Figure 13a); (**c**) average size statistics of precipitated phases; (**e**) HRTEM diagram; (**f**) electron diffraction patterns for selected areas.

Figure 13 presents the TEM images of the Cu-1.16Ni-0.36Cr alloy after aging for 4 h at 450 °C. Figure 13a shows that, compared with the alloy aged for 2 h at the same temperature, the dislocations in the alloy aged for 4 h at 450 °C are significantly reduced, and the dislocation cell size also increases. The figure shows that there is a clustering phenomenon of precipitates in the Cu matrix. Figure 13b presents the TEM image of the precipitates, which shows that the size of the precipitates is significantly larger than that after aging for 2 h, presenting a regular granular shape. At the same time, there is also a large amount of precipitate clustering at the grain boundaries, and the precipitates at the grain boundaries also have a significant increase in size compared to those in the matrix. Meanwhile, precipitates with a size of about 100 nm were observed. These are analyzed to be the growth of the primary phase, which are few in number. Selected-area electron diffraction analysis shows that they are body-centered cubic Cr precipitates, and the precipitate structure is stable. By statistically analyzing the size of the precipitates, it is found that after aging for 4 h at 450 °C, the size of the internal precipitates increases significantly, and the structure is more stable. At this time, the average grain size of the precipitates is 7.8 nm. Compared with the aging time, increasing the aging temperature has a more significant effect on the coarsening of the Cr precipitate size.

## 4. Analysis and Discussion

Aging parameters have a significant impact on alloy properties, primarily by altering microstructural features such as grain size, dislocation density, and the volume fraction and size of precipitated phases within the alloy. In this section, quantitative analysis of the alloy’s microstructural characteristics was conducted at different aging times under 450 °C, to explore the evolution of alloy strengthening mechanisms during aging.

### 4.1. Precipitation Kinetics

During aging, Cr elements in alloys gradually precipitate from the matrix. The solute content in the matrix and the volume fraction of precipitates significantly affect the alloy properties. Here, the precipitation kinetics during aging are described using the Avrami law [18].

(2) $$\varphi =1-{\mathrm{e}}^{-bt^{n}}$$

where φ is the precipitation fraction, defined as the ratio of the precipitate volume fraction at a given aging time to the maximum precipitate volume fraction, *t* is the aging time, and *b* and *n* are constants. Taking the logarithm of both sides of the equation yields

(3) $$lg\left(ln\frac{1}{1-\varphi }\right)=lgb+nlgt$$

**Figure 14:**
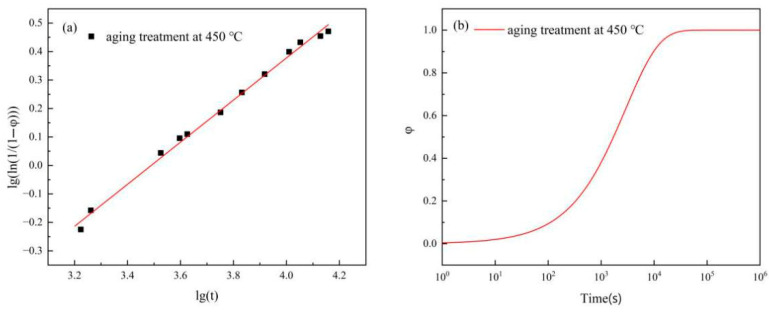
(**a**) *lg*(*ln*(1/(1 − *φ*))) − *lg*(*t*) linear fit chart; (**b**) precipitation volume versus time curve. Adapted from Ref. [17].

To obtain the precipitation fraction of the precipitated phase, the electrical conductivity of the alloy was tested at different aging times. In this section, it is considered that the electrical conductivity of the alloy is mainly affected by solute atoms [19]. As the aging time increases, the solute atoms in the alloy gradually precipitate in the form of precipitated phases, causing the electrical conductivity to gradually increase. After a sufficiently long aging time, the electrical conductivity of the alloy tends to stabilize, at which point it is considered that the precipitated phase of the alloy has completely precipitated. The precipitation fraction of the precipitated phase is calculated using the electrical conductivity at different aging times. In this experiment, only the precipitation of the chromium (Cr) phase was found, simplifying the situation. It can be considered that there is a linear relationship between the electrical conductivity and the precipitation fraction of the precipitated phase within the precipitated phase [20].

(4) $$\varphi =\frac{\xi -\xi_{0}}{\xi_{max}-\xi_{0}}$$

where ξ is the electrical conductivity of the Cu-Ni-Cr alloy at aging time *t*, $\xi_{0}$ is the electrical conductivity of the solution-treated alloy, with a measured value of 30.2% IACS, and $\xi_{max}$ is the maximum electrical conductivity of the annealed alloy, with a measured value of 48% IACS. The precipitation fraction of phases within the alloy at different aging times was determined by calculating the electrical conductivity. The scatter lg(ln(1/(1−φ)))−lgt plot was drawn, and the data were fitted using Equation (2). The results are shown in Figure 14a, where the intercept and slope of the resulting graph were *n* and *b*, respectively. These values were substituted into Equation (1), yielding the results presented in Figure 14b. The precipitation fraction of phases within the alloy at different aging times can be obtained through these equations.

As shown in Figure 14b, the increase in cold-rolling deformation promotes the precipitation of the precipitated phase in the later stage of aging. Next, we will calculate the volume fraction of the precipitated phase using Equation (5) [21].

(5) $$f=\frac{\sum \frac{m_{p}}{\rho_{p}}}{\frac{m_{Cu}}{\rho_{Cu}}+\sum \frac{m_{p}}{\rho_{p}}}$$

where $m_{p}$ is the mass when the precipitate phase is fully precipitated, $m_{Cu}$ is the mass of the copper matrix, and $\rho_{p}$ is the density of the precipitate phase, $\rho_{Cr}=7.19g/{cm}^{3}$, and $\rho_{Cu}=8.9g/{cm}^{3}$.

### 4.2. Dislocation Density

**Figure 15:**
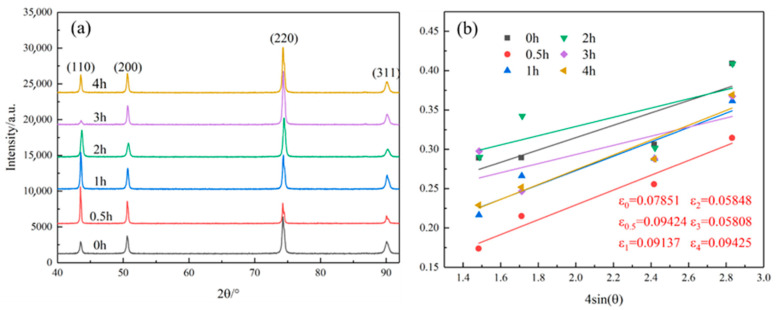
Aging alloys at different aging times. (**a**) XRD image; (**b**) linear fitting graph of 4sin(θ) − $\beta \cos\left(\theta \right).$

Figure 15 presents the XRD patterns of the alloy at different time points during the aging process. It can be observed from the XRD patterns that as the aging time increases, the diffraction peaks shift to the right, and no obvious new diffraction peaks emerge. The microstrain of the alloy is calculated using Equation (6) [22].

(6) $$\beta \cos\left(\theta \right)=\frac{k\lambda }{d}+4\varepsilon sin\left(\theta \right)$$

where β is the full width at half maximum of the diffraction peak, λ is the wavelength of the radiation, *k* is a constant with a value of 0.9, ε is the Bragg angle, and *d* is the grain diameter. Figure 15a presents the XRD patterns of the alloy under different cold-rolling deformation amounts. Figure 15b shows the $4sin\left(\theta \right)-\beta \cos\left(\theta \right)$ images of the alloy at different aging times. The experimental data were linearly fitted using Equation (6), yielding the slopes shown in the figure.

Therefore, the dislocation density of the alloy can be calculated based on the accumulated microstrain ε [23].

(7) $$\rho =\frac{2\sqrt{3}\varepsilon }{db}$$

where *b* is the Burgers vector and *d* is the grain diameter. The dislocation density within the alloy was calculated, with the results presented in Table 3, showing little change in dislocation density during the initial aging stage, a rapid decrease in dislocation density after 2 h of aging, and stabilization of dislocation density as aging time further increases.

### 4.3. Strengthening Mechanism

The mechanical properties of Cu-Ni-Cr alloy sheets were significantly enhanced after heat treatment, closely related to the precipitation of phases, evolution of dislocations, and grain structure during the deformation and heat treatment processes. By investigating the contributions of various strengthening mechanisms to the mechanical properties of Cu-Ni-Cr alloys, we can analyze the impact of cold-rolling reduction on the alloy during the thermomechanical treatment process. Four distinct strengthening mechanisms were considered in this study: solid-solution strengthening, precipitation strengthening, grain-boundary strengthening, and dislocation strengthening. The improvement in alloy strength can be viewed as the superposition of the contributions from these four strengthening mechanisms, which can be expressed by the following equation.

(8) $$\Delta \sigma_{\mathrm{alloy}}=\sigma_{0}+\Delta \sigma_{ss}+\Delta \sigma_{d}+\Delta \sigma_{GB}+\Delta \sigma_{p}$$

where $\Delta \sigma_{\mathrm{alloy}}$ is the strength of the aged Cu-Ni-Cr alloy and $\sigma_{0}$ is the strength of the copper matrix. Experimental results show that under the same testing conditions, the strength of as-cast pure copper is 131 MPa.

After solution treatment, alloying elements are incorporated into the copper matrix. Subsequent aging treatment causes most Cr elements to precipitate as precipitate phases, while Ni elements remain in solid solution form within the matrix. The presence of Ni and Cr atoms in solid solution within the matrix contributes to alloy strengthening. In this study, the Labusch theory [24] is employed for quantitative analysis of solid-solution strengthening in the aged Cu-Ni-Cr alloy.

(9) $$∆\sigma_{ss}=MG_{0}{\left[{\left(\beta \frac{a_{1}-a_{0}}{a_{0}}\right)}^{2}+{\left(2\frac{G_{1}-G_{0}}{G_{1}+G_{0}}\right)}^{2}\right]}^{\frac{2}{3}}c^{\frac{2}{3}}$$

where *M* is the Taylor factor equal to 3.0622 [25], *G*_0_ is the shear modulus of the copper matrix equal to 47 GPa, *a*_0_ is the lattice constant of the copper matrix equal to 0.361 nm, a1 is the lattice constant of the solute, Ni is 0.352, Cr is 0.288, *G*_1_ is the shear modulus of the solute atoms, Ni is 77 GPa, Cr is 115 GPa, and *c* is the concentration of solute atoms in the alloy [26,27]. According to the data in Table 3, the strengthening effect of solid-solution strengthening on the alloy at different aging times is shown in Table 4.

Dislocation strengthening is a mechanism where dislocations generated during plastic deformation enhance the strength of alloys. The higher the dislocation density, the greater the resistance to deformation, thereby enhancing the mechanical properties of the alloy. The contribution of dislocations to the strength of alloys can be estimated using the Bailey–Hirsch formula [28].

(10) $$∆\sigma_{d}=M\alpha Gb\rho^{\frac{1}{2}}$$

where *M* is the Taylor factor, α the constant for copper alloys equal to 0.2, *G* is the shear modulus of the copper matrix, *b* is the Burgers vector equal to $\sqrt{2}a/2$, taken as 0.255 nm, a is the lattice constant of the copper alloy, and ρ is the dislocation density [29]. As indicated by the dislocation density data in Table 3, the dislocation strengthening of the alloy under various aging times is presented in Table 4.

Grain boundaries strengthen materials by impeding dislocation motion. TEM microstructural analysis reveals that the relationship between precipitates and dislocations in this alloy follows an bypass mechanism. Grain-boundary strengthening can be quantified using the classical Hall–Petch equation [30].

(11) $$∆\sigma_{\mathrm{GB}}=кd^{-1/2}$$

where к is the Hall–Petch coefficient, different materials have different к values, and copper alloys have a value of 140 MPa ${\mu m}^{1/2}$. The grain size of the alloy obtained by EBSD is shown in Table 3, and the results of grain-boundary strengthening are shown in Table 4.

Precipitation has a significant effect on the strength of the alloy. During the deformation process, the deformation of the alloy is restricted by Cr particles, causing dislocation pile-up, which hinders alloy deformation and enhances its strength. As aging proceeds, the structure of Cr precipitates gradually stabilizes and loses the coherent relationship with the matrix, making the shearing of Cr particles difficult. The dispersed-dispersion phase enhances alloy strength through a pinning effect. Therefore, in this paper, the contribution of precipitation strengthening to strength is represented by the Orowan mechanism [31,32,33].

(12) $$∆\sigma_{p}=0.81\frac{MGb}{2\pi \sqrt{1-\upsilon }}\frac{ln(\bar{d}/b)}{\left(\lambda -\bar{d}\right)}$$

(13) $$\lambda =2r\left(\sqrt{\frac{\pi }{4f}}-1\right)$$

where *M* is the Taylor factor, υ is the Poisson’s ratio of the copper matrix equal to 0.34, *G* is the shear modulus of the matrix, *b* is the Burgers vector, $\bar{r}$ is the average radius of the precipitate phase, $\bar{r}$ = $\sqrt{2∕3}r$, $\bar{d}$ is the average diameter of the precipitate phase, λ is the distance between the edges of the precipitate phase, and λ can be obtained from Equations (3)–(12), where f is the volume fraction of the precipitate phase. The precipitation strengthening of the alloy at different aging times is shown in Table 4.

**Figure 16:**
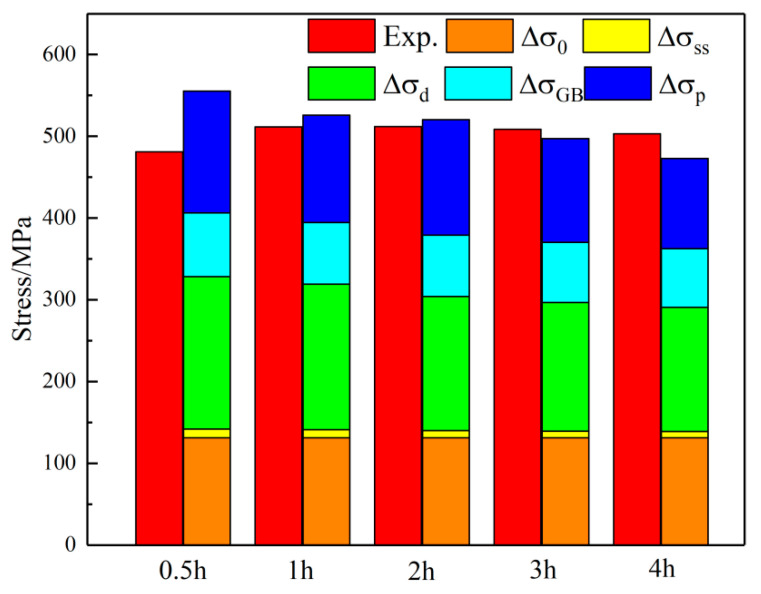
Contribution diagram of various strengthening mechanisms to strength of Cu-Ni-Cr alloy aged at 450 °C.

The strength of the alloy after aging at 450 °C for different times and the contributions of each strengthening mechanism are shown in Figure 16. The results indicate that this alloy is a typical precipitation-strengthened copper alloy, with significant strengthening from precipitates relative to the copper matrix. As aging time increases, the volume fraction of precipitates rises, promoting precipitation strengthening. However, as aging time extends, precipitate size increases. Excessive aging time reduces alloy strength. Research indicates that aging at 450 °C for 2 h optimizes precipitate size and volume fraction, yielding the best comprehensive alloy properties. Severe deformation introduces numerous dislocations, which hinder grain deformation and significantly enhance alloy strength. Grain refinement from severe deformation also moderately improves alloy strength. Ni has a lattice constant close to the matrix, Cr has a high precipitation ratio, and the alloy content is low, so solid-solution strengthening contributes little to strength enhancement.

## 5. Conclusions

1. The deformation heat treatment process significantly enhances the mechanical properties and electrical conductivity of Cu-1.16Ni-0.36Cr alloy. After undergoing forging + solution treatment + cold rolling + annealing + second cold rolling + 2 h aging at 450 °C, this alloy develops a large number of Cr precipitates, with a BCC structure and about 10 nm in size, which are dispersed in the alloy matrix. As a result, it achieves excellent comprehensive properties, with a strength of 512 MPa, elongation of 17.2%, and electrical conductivity of 45.5% IACS.

2. Aging treatment promotes the precipitation and growth of nanoscale Cr precipitates. The increase in aging temperature provides more energy for the precipitation of Cr precipitates, thereby promoting their precipitation and growth. When the aging temperature is increased from 400 °C to 450 °C, the structure of Cr precipitates gradually stabilizes, exhibiting granular body-centered cubic (BCC) structured Cr precipitates. When the aging temperature is further increased to 500 °C, the size of Cr precipitates begins to increase significantly, with the average size growing from 5.0 nm to 16.7 nm. The increase in aging time results in a slight increase in the volume and size of Cr precipitates.

3. The internal microstructural characteristics of the alloy aged at 450 °C for different durations were summarized, and the precipitation characteristics of the precipitated phase and the contribution of the strengthening mechanism to the alloy strength were analyzed, establishing the intrinsic relationship between aging parameters, microstructural characteristics, strengthening mechanisms, and mechanical properties. The results show that the strengthening mechanisms of the alloy are mainly precipitation strengthening and grain-boundary strengthening. With the increase in aging time, grain-boundary strengthening shows a gradually decreasing trend. In the early stage of aging, due to the increase in precipitation degree and the stabilization of the precipitated phase structure, the precipitation strengthening effect gradually increases. As aging proceeds, the precipitated phase coarsens, and the precipitation strengthening effect weakens. At peak aging, grain-boundary strengthening accounts for 32.0%, and precipitation strengthening accounts for 27.5%.

## Figures and Tables

**Table 1:** Strength and elongation of alloys under different aging parameters.

Time/h	400 °C		450 °C		500 °C	
	Tensile Stress/MPa	Elongation/%	Tensile Stress/MPa	Elongation/%	Tensile Stress/MPa	Elongation/%
0.5 h	427.5	12.5	481.0	13.4	465.6	14.0
1 h	426.9	13.8	511.4	14.8	454.4	19.6
2 h	453.2	17.3	512.0	17.2	448.7	19.5
3 h	464.9	17.0	508.6	12.1	438.5	18.5
4 h	475.2	18.0	503.0	12.4	420.3	18.0

**Table 2:** Texture statistics.

	Temperature /°C	0	400 °C	450 °C	500 °C
Type					
Cube		14.5	2.3	5.8	2.5
Goss		2.2	9.0	3.3	3.0
Brass		31.7	29.4	12.1	29.2
Copper		24.5	28.6	38.8	41.5
S		61.4	48.9	47.2	27.3

**Table 3:** Model parameter.

Aging Time/h	Elemental Cr Solute Concentration/%	Cr Precipitation Phase Volume Fraction/%	Dislocation Density/×10^14^/mm	Grain Size/μm	Average Size of Precipitated Phase/nm
0.5	0.165	0.24	6.45	3.20	3.2
1	0.110	0.31	5.90	3.44	4.8
2	0.039	0.40	5.00	3.46	5.0
3	0.016	0.43	4.60	3.65	6.2
4	0.004	0.45	4.30	3.95	7.8

**Table 4:** Enhanced contribution.

Aging Time/h	Solid-Solution Strengthening/MPa	Grain-Boundary Strengthening/MPa	Dislocation Strengthening/MPa	Precipitation Strengthening/MPa
0.5	11.0	78.1	186.3	148.8
1	10.0	75.4	178.2	131.3
2	9.0	75.3	164.0	141.0
3	8.4	73.6	157.3	126.9
4	7.8	71.7	152.1	110.4

## Data Availability

The original contributions presented in the study are included in the article; further inquiries can be directed to the corresponding author.
